# Pediatric Clinical Trials in Mainland China Over the Past Decade (From 2009 to 2020)

**DOI:** 10.3389/fmed.2021.745676

**Published:** 2021-10-04

**Authors:** Wen-Wen Wu, Xing Ji, Hao Wang, Feng Chen, Qian Ding, Guan-dong Zhang, Man Li, Shan-shan Wang, Ming-ming Ni, Qing-qing Liu, Jing Xu, Qian Wang

**Affiliations:** ^1^Department of Pharmacy, Children's Hospital of Nanjing Medical University, Nanjing, China; ^2^Nanjing Drum Tower Hospital, The Affiliated Hospital of Nanjing University Medical School, Nanjing, China; ^3^Clinical Research Center, National Center for Children's Health, Beijing Children's Hospital, Capital Medical University, Beijing, China; ^4^Office of Clinical Trial Institution, Shanxi Children's Hospital, Taiyuan, China

**Keywords:** pediatric clinical trials, National Medical Products Administration, Chinese Clinical Trials Registry and Information Transparency Platform, pediatric population, drug research and development

## Abstract

In mainland China, there remains a shortage of pediatric drugs. The Chinese government has recently launched policies and incentives to encourage pediatric drug development and clinical trials. However, data on the characteristics or development trends of these trials are limited. In this review, we extracted source data from the Chinese Clinical Trials Registry and Information Transparency Platform and systematically reviewed the pediatric clinical trials conducted in mainland China from 2009 to 2020, a comprehensive process evaluation of the pediatric drug clinical trials development in the past decade, providing data support to policy makers and industry stakeholders. We included 487 pediatric clinical trials. Over the past decade, the number of pediatric trials has increased, especially since 2016. The most common therapeutic areas were infectious diseases (*n* = 108, 22.2%), agents for preventive purpose (*n* = 99, 20.3%), and neurological and psychiatric diseases (*n* = 71, 14.6%). The number of clinical trials involving epilepsy (39, 10.1%), asthma (33, 8.5%), and influenza (24, 6.2%) were the highest. The distribution of leading institutions is unbalanced in mainland China, with most units in East China (34.0%) and few in Southwest China (6.9%). China has made progress in improving the research and development environment of pediatric drugs and increasing pediatric trials. However, a wide gap in pediatric drug development and clinical trials quality exists between China and the developed countries. The pharmaceutical industry in China has faced grim setbacks, including study duplication, lack of innovation, poor research design, and unbalanced resource allocation. Thus, we suggest that the Chinese government should adjust their policies to improve innovation and clinical design capacity, and optimize resource allocation between regions.

## Introduction

According to the statistics of the UN Children's Fund, the pediatric population aged 0–17 in China was 217 million in 2015, accounting for 13.0% of the world total and ranking in second place. Promoting safe drug use and protecting children's health are long-term health goals in China. To protect children's health, it is necessary to first meet their medicinal needs. In China, however, the shortage of pediatric drugs is severe. Data in the White Paper on the Investigation of Pediatric Drug Use Safety (1) released in 2016 show that, of the 176,652 drugs approved in China by June 2016, only 3,517 were pediatric drugs, accounting for <2.0%. Thus, it is crucial to deal with the issue at a policy level, encouraging the development of pediatric drugs and their relevant clinical trials, and promoting the approval and marketing of pediatric drugs.

Over the past decade, especially since 2014, the Chinese government has launched a package of policies to encourage the development of pediatric drugs. However, research focusing on the current situation, the characteristics of pediatric trials, and their development trends is relatively rare.

At present, all the studies on pediatric clinical trials in mainland China that we have retrieved are based on data registered in the Chinese Clinical Trial Registry (Chi CTR) (2–4).

The Chi CTR database is not compulsorily collected for drug approval and marketing and focuses on investigator-initiated clinical studies rather than new drug development, limiting its use in analyzing the current situation of pediatric drug development in China.

Therefore, this study used the Chinese Clinical Trials Registry and Information Transparency Platform (CCTR and ITP) for source data. This platform was established in 2013 by the former China Food and Drug Administration (CFDA) and requires all entities who have been granted clinical trial approval and plan to conduct trials in China to register and make the information regarding the clinical trials public. With an in-depth analysis into the data on the platform through longitudinal and horizontal comparison of quantitative and qualitative indicators in different dimensions and a comprehensive process evaluation of the pediatric drug clinical trials development in the past decade, we hope to shed light on the current situation and development trends of pediatric clinical trials and detect existing problems. The findings of this analysis will provide effective data support for policy makers and other stakeholders.

## Retrieval Strategy and Selection Standard

In 2013, the former CFDA, in accordance with World Health Organization requirements and international conventions, established the CCTR and ITP and stipulated that all entities who have been granted clinical trial approval and plan to conduct the trials [including bioequivalence (BE) studies, pharmacokinetics tests, and Phase I, II, III, and IV trials] in China must register on the platform. To ensure data timeliness, the initial registration must be submitted at least 30 days before the first subject is enrolled. The CFDA cross-checks all submitted clinical reports and materials on a regular basis to ensure data validity and integrity. In addition, clinical trials started before 2013 that have not received sales and marketing approval are required to register retrospectively.

The information made public on the platform includes information on sponsors and investigators, the first registration date, basic information regarding the clinical trial (drug name, indication, trial purpose, and trial design), subject information (inclusion and exclusion criteria and the number of subjects), primary and secondary endpoint criteria, ethical review, trial development, and a summary of the results.

**Figure 1:**
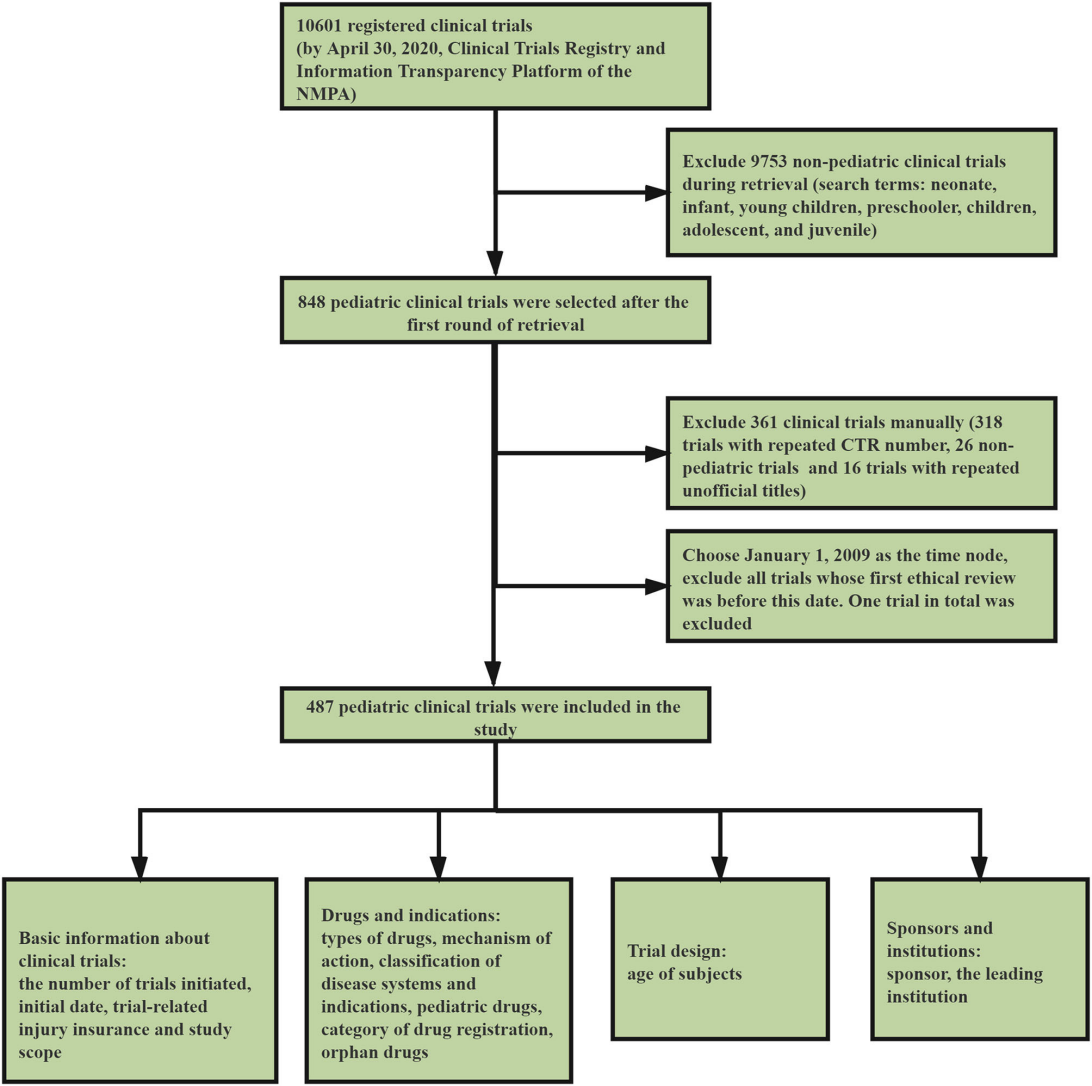
Strategy of retrieval and selection of pediatric clinical trials.

We conducted a systematic review of the clinical trial information submitted to the platform before April 30, 2020. After preliminary retrieval, 10,601 registered clinical trials were found (Figure 1). Our study involved all the pediatric clinical trials, and the retrieval and data-processing was conducted in three steps. The first step was to select clinical trials which involved pediatric indications and conducted by institutions specializing in pediatric drugs. We used the search terms *neonate, infant, young children, preschooler, children, adolescent*, and *juvenile*. After the first round of screening, 848 clinical trials were incorporated into the study (Figure 1).

In the second step, we used Excel to screen and delete 318 trials with repeated Clinical Trials Register Numbers and remove 16 trials with repeated unofficial titles. Next, two pediatricians were invited to independently review the selected clinical trials and, if there was any disagreement, a third pediatrician was asked to arbitrate until an agreement was reached. This step helped to exclude 46 non-pediatric trials. To ensure that no relevant trials were omitted, we employed simple random sampling and selected 10.0% of the trials from the excluded group (*n* = 10,115). The selected 10.0% of trials were then reviewed by the two pediatricians mentioned above, and no pediatric-relevant trials were found.

The year 2020 has witnessed the ending of the National Major Scientific and Technological Special Project for “Significant New Drugs Development.” Launched in 2008, the project symbolized the start of a new era for drug development in China. We selected January 1, 2009, just 1 year after the launching of the project, as the time node, and excluded all trials which underwent initial ethical review before this date. Only one trial that received its ethical review in 2005 was excluded. Finally, 487 pediatric clinical trials were included in this study. The third step involved data preparation and statistics. We statistically classified and analyzed the selected clinical trials according to various dimensions, including basic information on trials, drugs, indications, trial design, sponsors, and institutions.

## Data Analysis Methods

We employed Excel and SPSS to process and analyze the data, adopting descriptive analyses and using percentages to indicate qualitative variables. All data were collected and analyzed according to the four dimensions: basic clinical trial information, drugs and indications, trial design, sponsor and institution. To evaluate the development process of pediatric drug clinical trials in the past decade, we further subdivided the statistical data as follows: the trend of the quantity was reflected by the number of clinical trials for different drugs, drugs in different study phases, and initial registration date and development trends. For clinical trial quality, we used disease system classification and indications and drug registration types to reflect the innovation aspect of the clinical trial. Trial-related injury insurance reflected the clinical trial design normalization. Study scope reflected the clinical trial impact. Clinical trials of pediatric and orphan drugs reflected the specificity of pediatric clinical trials. The above indicators reflect the quality trend of pediatric clinical trials in China. Finally, the resource allocation of pediatric clinical trials was reflected by clinical trial sponsors and institutions. A simple regression model was used to calculate the annual fluctuation rates of the indicators. The homogeneity of variance test was used to compare whether the same index had statistical differences under different statistical diameters. The initial year of the trial was determined by the date of its first ethical review. Two-tailed *P* < 0.05 were considered significant.

## Results

### Basic Information Regarding Pediatric Clinical Trials in China

#### General Conditions

**Figure 2:**
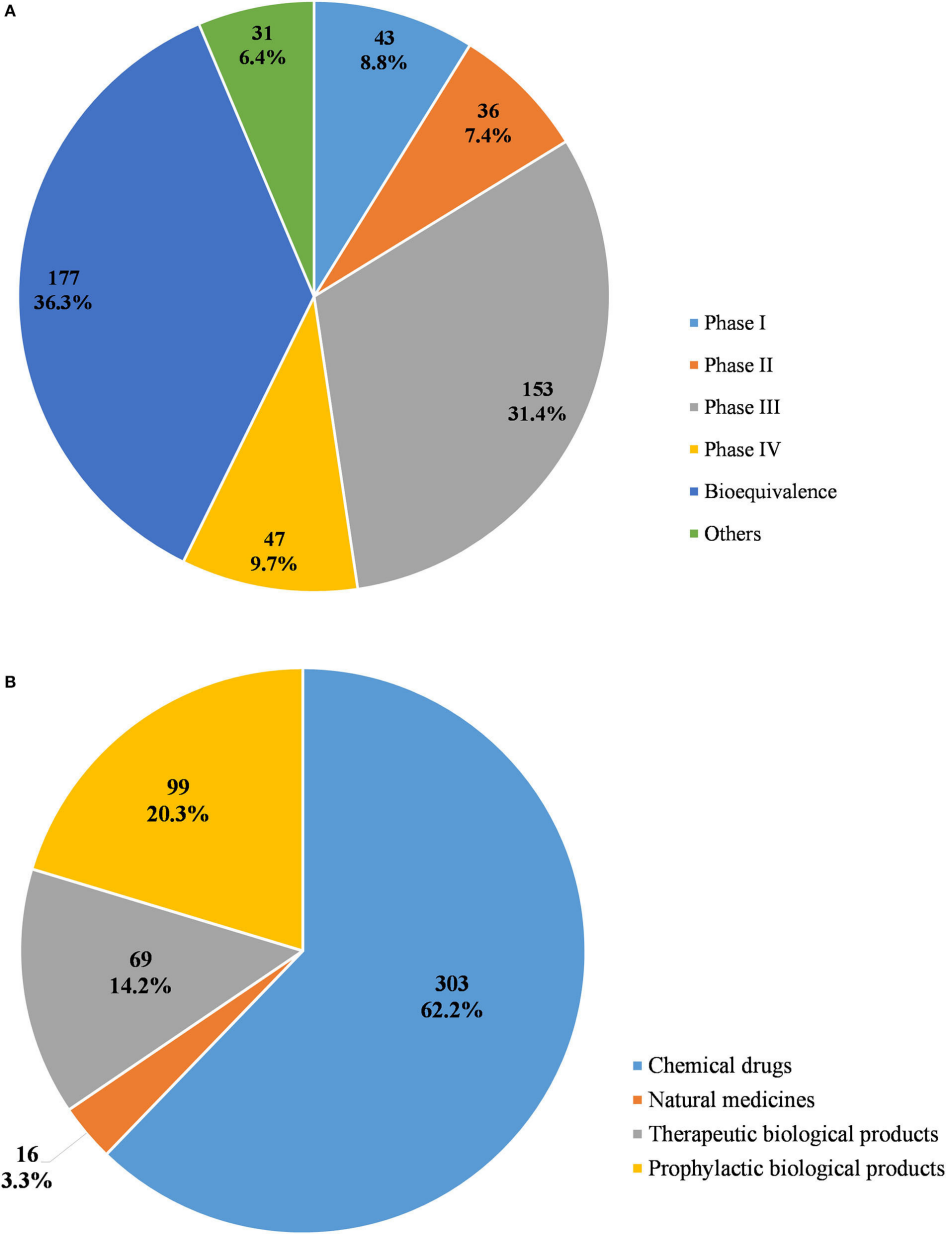
Distribution of pediatric clinical trials by classification of study phases **(A)** and drug type **(B)**.

From 2009 to April 2020, a total of 487 pediatric clinical trials were conducted in mainland China, of which 246 (50.5%) were ongoing, 226 (46.4%) had been completed, and 15 (3.1%) had been voluntarily suspended by their sponsors. Of the 487 trials, 43 were in Phase 1 (8.8%), 36 in Phase II (7.4%), 153 in Phase III (31.4%), and 47 in Phase IV (9.7%), 177 (36.3%) were BE studies, and the last 31 (6.4%) fell into other categories (Figure 2A). When classified in terms of drug type, chemical drugs were involved in 303 trials (62.2%); natural medicines in 16 trials (3.3%); therapeutic biological products in 69 trials (14.2%), and prophylactic biological products in 99 trials (20.3%) (Figure 2B).

#### Initial Registration Date and Development Trends

**Figure 3:**
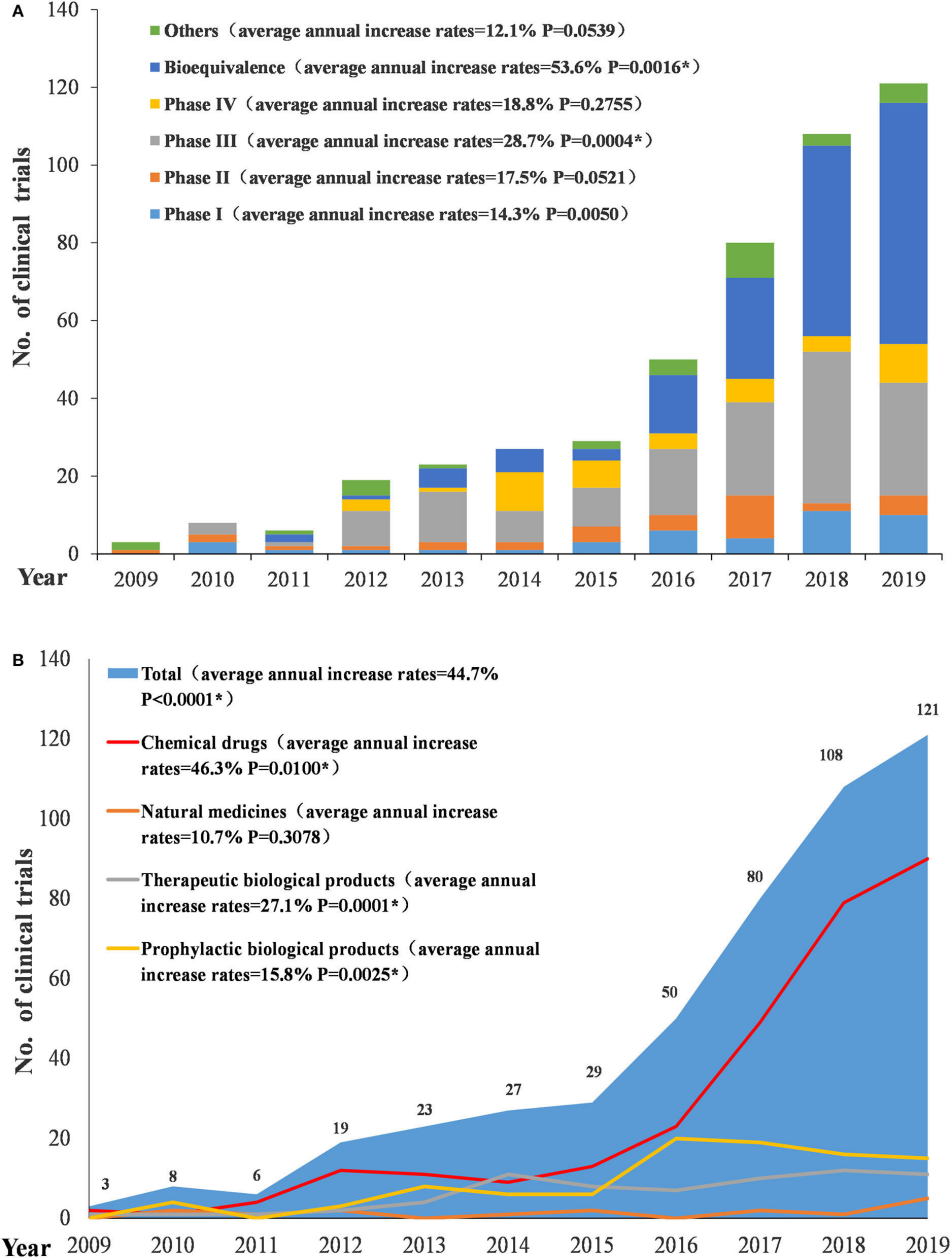
Number and initial time of pediatric clinical trials by drug type **(A)** and study phase **(B)**.

From 2009 to 2019, the number of conducted trials increased annually (*P* < 0.0001), with an average annual increase rate of 44.7%. Since 2016, the number of pediatric-related clinical trials began to rise significantly, with an annual increase of more than 20 trials. The increase was significant in 2017 and 2018 with an annual increase of 30 trials. Conversely, in 2019, the increase began to slow. In terms of the types of drugs involved in pediatric trials, chemical drugs showed a significant increase, with an average annual increase rate of 46.3% (*P* = 0.01); natural medicines, therapeutic biological products, and prophylactic biological products showed relatively small changes, with average annual increase rates of 10.7% (*P* = 0.3078), 27.1% (*P* = 0.00012), and 15.8% (*P* = 0.0025), respectively, between 2010 and 2019.Trials on both chemical drugs and prophylactic biological products began to significantly increase in 2016 (Figure 3A). In terms of study phases, there were few Phase I, II, and IV trials, with little fluctuation, while Phase III trials and BE studies both saw significant increases in 2016. Phase III trials had an average annual increase rate of 28.7% (*P* = 0.0004) (2010–2019) and BE studies had an average annual increase rate of 53.6% (*P* = 0.0016) (2011–2019) (Figure 3B).

#### Trial-Related Injury Insurance

Of the 487 clinical trials, 218 provided trial-related injury insurance to the participants, with an overall coverage rate of 44.8%. The coverage rate of the international multi-center trials, however, was 88.5%, much higher than that of the domestic trials (8.5%) (F = 54.0, *P* < 0.001) (Table 1). From 2010 to 2019, the insurance coverage rate of pediatric clinical trials in China showed an upward trend, with an average annual increase rate of 35.3% (*P* = 0.0019). The highest insurance coverage rate was observed in Phase III trials (57.5%), followed by BE trials (41.2%). The insurance coverage rates of Phase I, II, and IV trials were 39.5, 33.3, and 36.2%, respectively (Table 2).

**Table 1:** Domestic and international multi-center trial-related injury insurance.

**Trial scope**	**No. of injury insurance trials (each scope total trials)**	**%**
Domestic	164 (426)	38.5
International multi-center	54 (61)	88.5
Total	218 (487)	44.8

**Table 2:** Different phase trial-related injury insurance.

**Trial phase**	**No. of injury insurance trials (each phase total trials)**	**%**
Phase I	17 (43)	39.5
Phase II	12 (36)	33.3
Phase III	88 (153)	57.5
Phase IV	17 (47)	36.2
BE study	73 (177)	41.2
Others	11 (31)	35.5
Total	218 (487)	44.8

#### Study Scope

Of the 487 trials, 258 were multi-center trials (52.3%) including 61 international multi-center trials (12.5%). The number of international multi-center trials increased over time, with an average annual increase rate of 37.9% (*P* = 0.0069) from 2012 to 2019. While the average number of international multi-center trials conducted per year from 2009 to 2016 was only 1.2, and drastically increased to 17 from 2017 to 2019. International multi-center trials began to increase significantly in 2017. In terms of study phases, Phase III trials contributed the highest proportion of international multi-center trials, with 49 trials accounting for 32.0%.Phase II trials ranked second, with seven trials accounting for 19.4% (Table 3). In terms of drug type, trials on chemical drugs contributed the highest proportion of international multi-center trials, with 54 trials accounting for 17.8%. In addition, six international multi-center trials on therapeutic biological products were registered, accounting for 8.7%. There was only one international multi-center trial on prophylactic biological products (1.0%) and no international multi-center trials on natural medicines (Table 4).

**Table 3:** Different trial phase scope.

**Trial phase**	**No. of Domestic trial (%)**	**No. of international multi-center trial (%)**	**Total no. of trials**
Phase I	43 (100.0)	0 (0.0)	43
Phase II	29 (80.6)	7 (19.4)	36
Phase III	104 (68.0)	49 (32.0)	153
Phase IV	46 (97.9)	1 (2.1)	47
BE study	175 (98.9)	2 (1.1)	177
Other	29 (93.5)	2 (6.5)	31
Total	426 (87.5)	61 (12.5)	487

**Table 4:** Scope of trials for different drug types.

**Drug type**	**No. of Domestic trial (%)**	**No. of international multi-center trial (%)**	**Total no. of trials**
Chemical drugs	249 (82.2)	54 (17.8)	303
Natural medicines	16 (100.0)	0 (0.0)	16
Therapeutic biological products	63 (91.3)	6 (8.7)	69
Prophylactic biological products	98 (99.0)	1 (1.0)	99
Total	426 (87.5)	61 (12.5)	487

#### Age of Subjects

**Figure 4:**
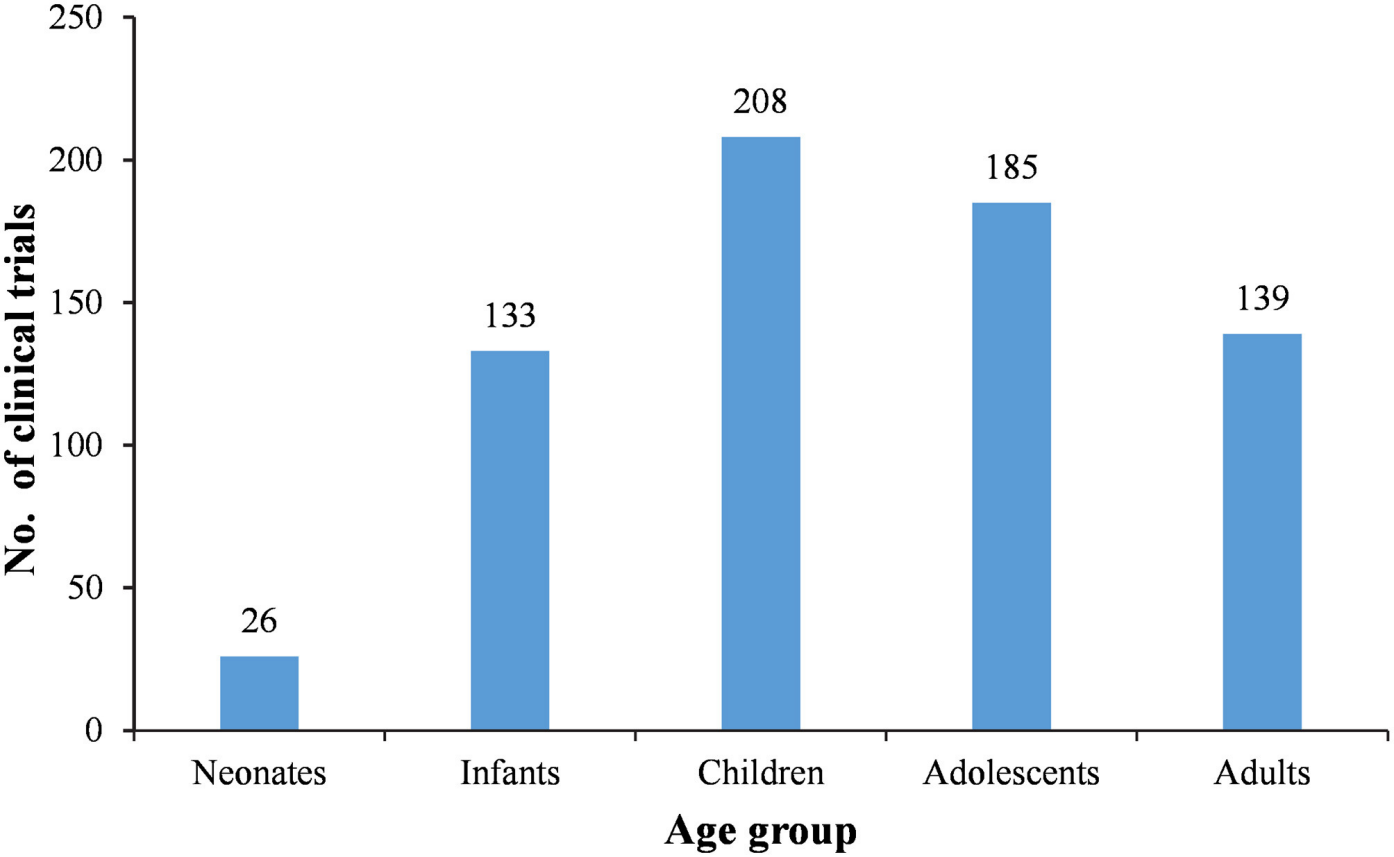
Distribution of pediatric clinical trials by age group.

Since it is a design requirement that all BE study subjects should be adults, the 177 BE studies were first excluded. Then, subjects were divided into five age groups, neonates (age <28 days), infants (28 days < age <24 months), children (2 years < age <11 years), adolescents (12 years < age <18 years), and adults (age > 18 years). After excluding the BE studies, 27 of the remaining 310 trials involved adult subjects only, accounting for 8.7%. The remaining 91.3% involved subjects <18 years of age, while 55.2% (171 trials) only involved subjects <18 years of age (Table 5). More specifically, 26, 133, 208, 185, and 139 trials involved neonate, infant, children, adolescent, and adult subjects, respectively (Figure 4).

**Table 5:** Distribution of clinical trials by different age groups.

**Age groups of subjects**	**No. of trials**	**%**	
Subjects included adults	Adults	27	8.7
	neonates, infants, children, adolescents, adults	13	4.3
	infants, children, adolescents, adults	23	7.4
	children, adolescents, adults	41	13.2
	adolescents, adults	35	11.3
Subjects included no adults	infants	32	10.3
	infants, children	38	12.3
	infants, children, adolescents	19	6.1
	neonates	5	1.6
	neonates, infants	2	0.6
	neonates, infants, children	2	0.6
	neonates, infants, children, adolescents	4	1.3
	children	19	6.1
	children, adolescents	49	15.8
	adolescents	1	0.3
Total	310	1	

### Drugs and Indications

#### Classification of Disease Systems and Indications

**Figure 5:**
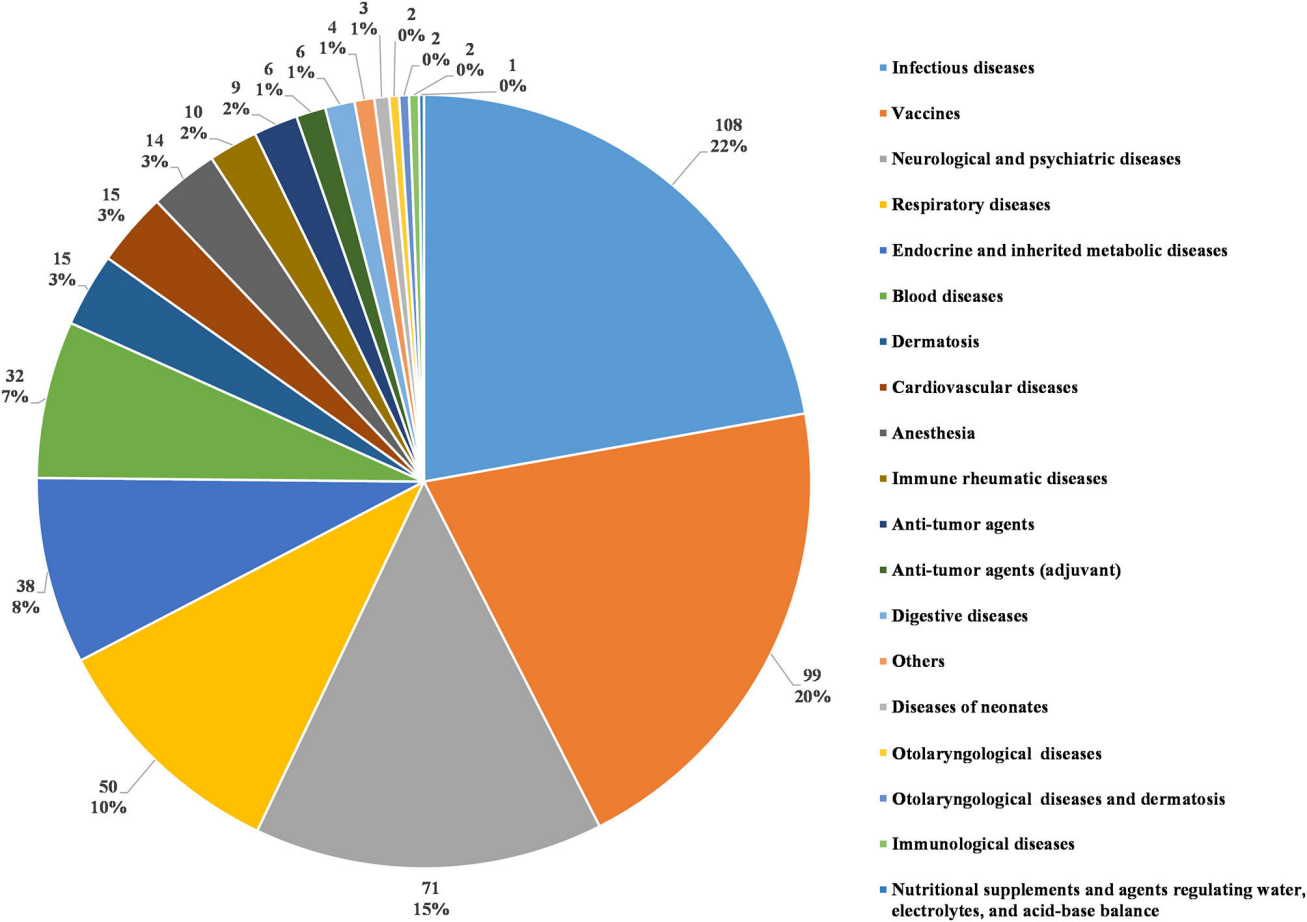
Distribution of pediatric clinical trials by disease system.

In accordance with the classification standards in the *Chinese National Formulary*, we classified all investigational products in terms of disease systems and indications (5). The number of trials for each classification was calculated (as some investigational products were tested for several indications, the number of investigational products classified in this way was higher than the actual number of tested products). Infectious disease was the most studied therapeutic area, with 108 clinical trials (22.2%). The next four most common therapeutic areas, in descending order, were agents for preventive purposes (99 trials, 20.3%), neurological and psychiatric diseases (71 trials, 14.6%), respiratory diseases (50 trials, 10.3%), and endocrine and inherited metabolic diseases (38 trials, 7.8%). Trials involving the five disease systems mentioned above accounted for 75.2% of all pediatric clinical trials (Figure 5).

As for indications, the trials on prophylactic biological products were first excluded, hence 388 trials remained, of which indications investigated in more than 10 trials included epilepsy (39 trials, 10.1%), asthma (33 trials, 8.5%), influenza (24 trials, 6.2%), growth retardation in children (24 trials, 6.2%), HIV infection/chronic hepatitis B (22 trials, 5.7%), HIV infection (19 trials, 4.9%), bacterial infectious diseases (14 trials, 3.6%), anesthesia (14 trials, 3.6%), hemophilia A (13 trials, 3.4%), chronic hepatitis B (11 trials, 2.8%), immune rheumatic diseases (10 trials, 2.6%), and attention deficit hyperactivity disorder (10 trials, 2.6%). In total, 233 trials involved the indications mentioned above, accounting for 60.0% of the 388 trials. There are 22 kinds of indications involving more than five clinical trials, the number of clinical trials is 299, accounting for 77.1% (Table 6).

**Table 6:** Distribution of clinical trials by indications.

**Indications**	**No. of trials**	**%**
Epilepsy	39	10.1
Asthma	33	8.5
Influenza	24	6.2
Growth retardation in children or microsomia	24	6.2
HIV infection/chronic hepatitis B	22	5.7
HIV infection	19	4.9
Bacterial infectious diseases	14	3.6
Anesthesia	14	3.6
Hemophilia A	13	3.4
Chronic hepatitis B	11	2.8
Immune rheumatic diseases	10	2.6
Attention deficit hyperactivity disorder	10	2.6
Respiratory tract infections in children	9	2.3
Mucous sputum of respiratory tract	8	2.1
Hyperlipidemia	8	2.1
Acute lymphoblastic leukemia	7	1.8
Common cold and fever	6	1.5
Cough in children	6	1.5
Atopic dermatitis	6	1.5
Hemophilia	6	1.5
Adjuvant chemotherapy	5	1.3
Schizophrenia or bipolar disorder	5	1.3
**Total**	**299**	**77.1**

#### Classification System for Registration of Chemical Drugs in China

In accordance with the Reform Scheme of the Classification System for Registration of Chemical Drugs issued by the CFDA in March 2016, and the Provisions for Drug Registration, which came into force on July 1, 2020, the chemical drugs were classified into innovative drugs, modified new drugs, and generic drugs (6, 7) (Table 7).

**Table 7:** Classification system for registration of chemical drugs.

**Classification**	**New classification**	**Definition/scope**	**Old classification**	**Definition/scope**
Innovative drugs	Class 1	Innovative drugs which have never been marketed within or outside China: compounds with clear and new chemical structures; with pharmacological effects and clinical value, that can be put into clinical practice.	Class 1, 1.1	Drugs not marketed within or outside China: drug substances and preparations produced by synthesis or semi-synthesis methods.
			Class 1, 1.2	Drugs not marketed within or outside China: new effective monomers and their preparations extracted from natural materials or by fermentation.
Modified new drugs	Class 2, 2.1	Drug substances and preparations with obvious clinical advantages: optical isomers of known active ingredients, optical isomers produced by splitting or synthesis methods; esterification or salt formation of known active ingredients (including salts containing hydrogen bonds or coordination bonds); modification of acid radical, base, or metal elements of known active ingredients of salt drugs; or other non-covalent bond derivatives (such as complex, chelates, or inclusion compounds).	Class 1, 1.3	Drugs not marketed within or outside China: optical isomers of known drugs and preparations; optical isomers produced by resolution or synthesis methods.
			Class 4	Drug substances and preparations with modifications of acid radical, base, or metal elements of already marketed salt drugs, without influencing their pharmacological effects.
	Class 2, 2.2	Preparations of known active ingredients with new dosage forms (including new drug delivery systems), formulations or routes of administration; with obvious clinical advantages.	Class 2	Preparations not marketed within or outside China: with changes to route of administration.
			Class 5	Preparations with changes to the dosage form of products already marketed in China; no changes to routes of administration.
	Class 2, 2.3	New compound preparations of known active ingredients, with obvious clinical advantages.	Class 1, 1.4	Drugs not marketed within or outside China: fewer-component drugs obtained from already marketed multi-component drugs.
			Class 1, 1.5	Drugs not marketed within or outside China: new compound preparations.
	Class 2, 2.4	Preparations of known active ingredients, with new indications.	Class 1, 1.6	Preparations already marketed in China, with new indications added; new indications previously unapproved within or outside China.
Generic drugs	Class 3	Drug substances and preparations with the identical active ingredients, dosage form, strength, indications, route of administration, and dosage to innovative drugs that have been marketed outside China but not in China.	Class 3, 3.1	Drug substances and preparations marketed outside China, and/or preparations with changes to dosage form but not route of administration.
			Class 3, 3.2	Compound preparations marketed outside China, and/or preparations with changes to dosage form but not route of administration.
			Class 3, 3.3	Preparations marketed outside China, with changes to route of administration.
			Class 3, 3.4	Preparations already marketed in China, with new indications added; new indications already approved outside China.
	Class 4	Drug substances and preparations with the identical active ingredients, dosage, strength, indications, route of administration, and dosage to innovative drugs that have been marketed in China.	Class 6	Drug substances or preparations which already have national standards.
Imported innovative drugs	Class 5, 5.1	Imported innovative drugs (including drug substances and preparations) that have been marketed outside China.		
Imported non-innovative drugs	Class 5, 5.2	Imported non-innovative drugs (including drug substances and preparations) that have been marketed outside China.		

**Figure 6:**
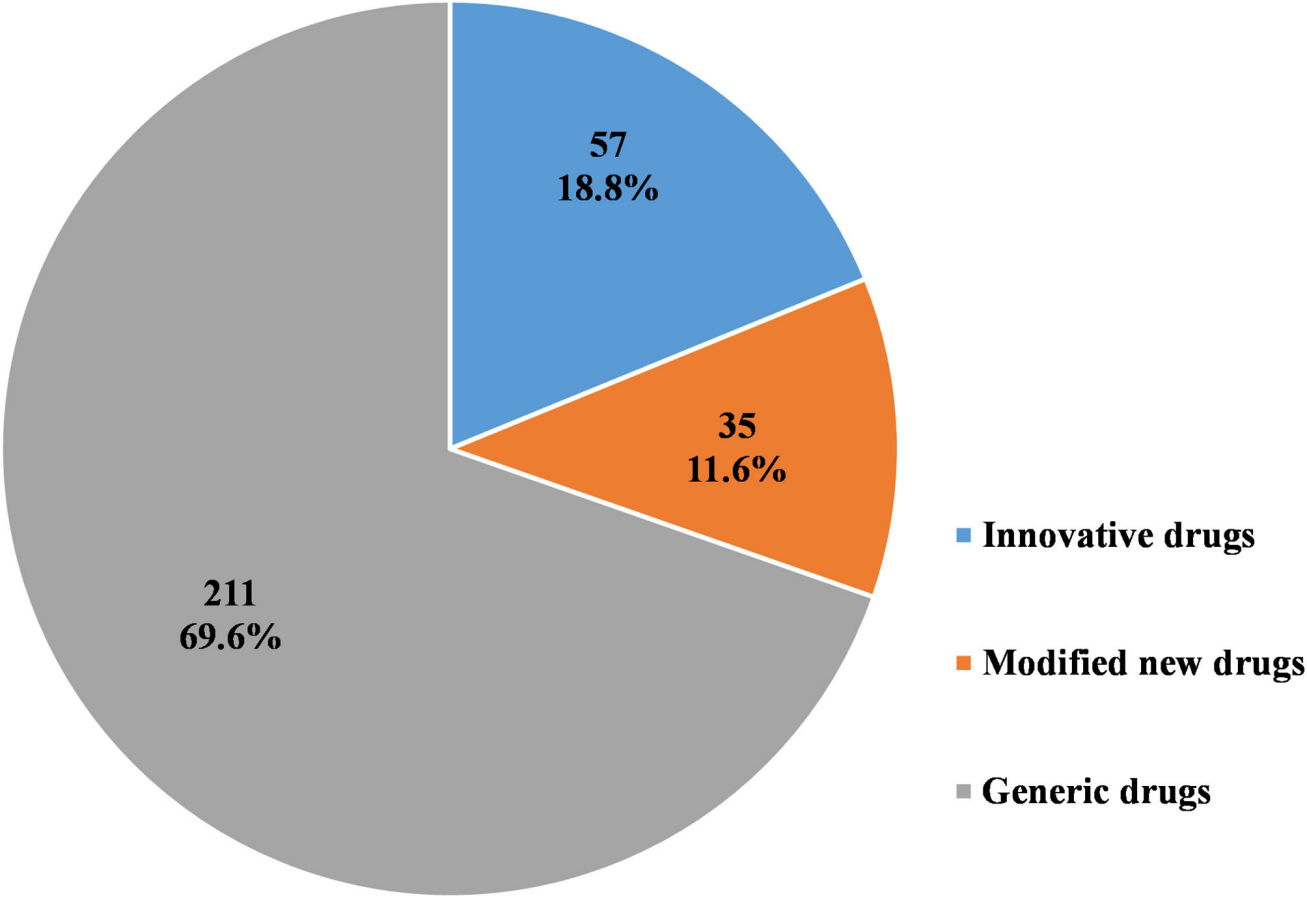
Distribution of chemical drugs by registration classification in pediatric clinical trials.

**Figure 7:**
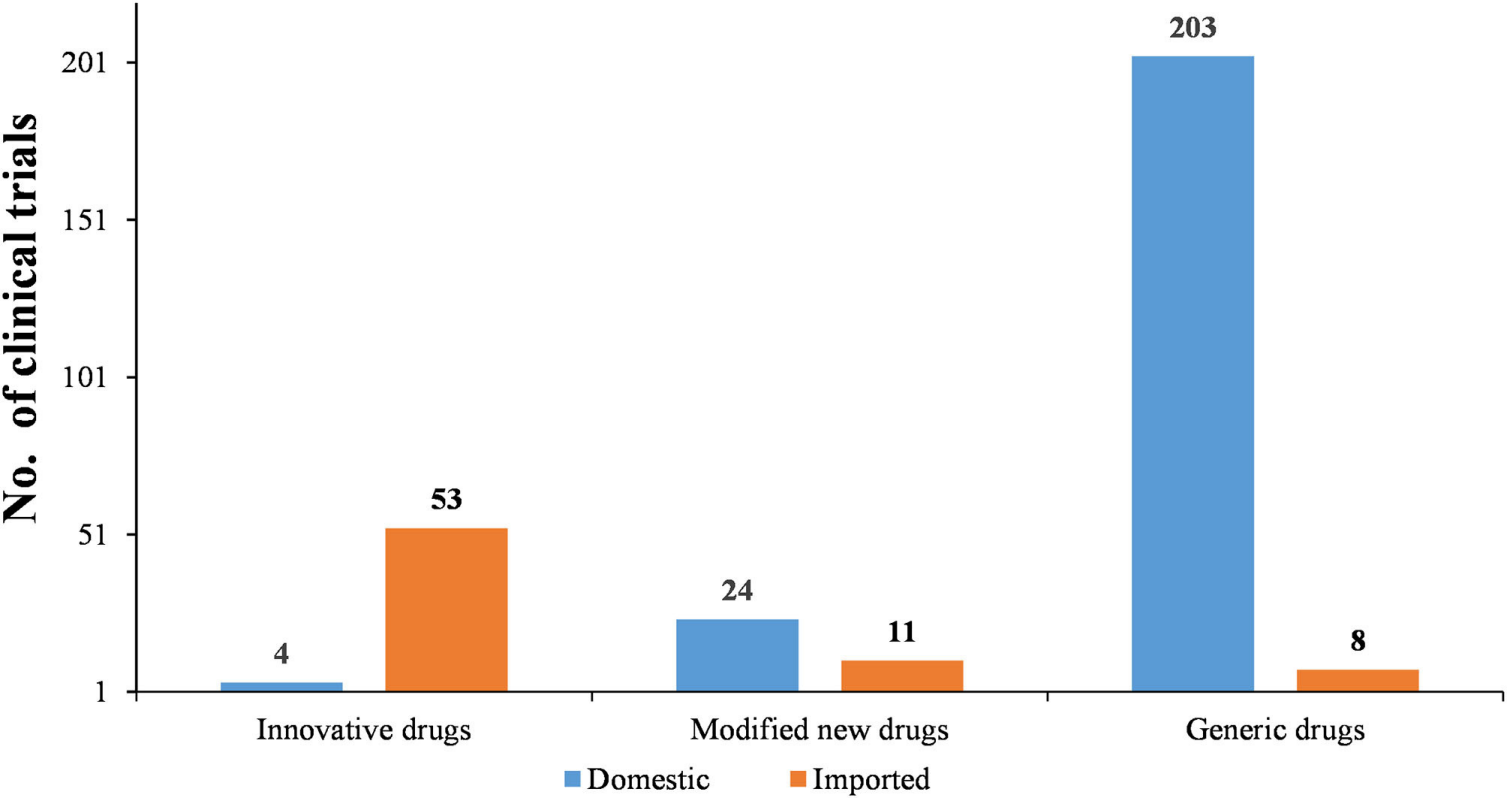
Distribution of domestic and imported chemical drugs in pediatric clinical trials.

**Figure 8:**
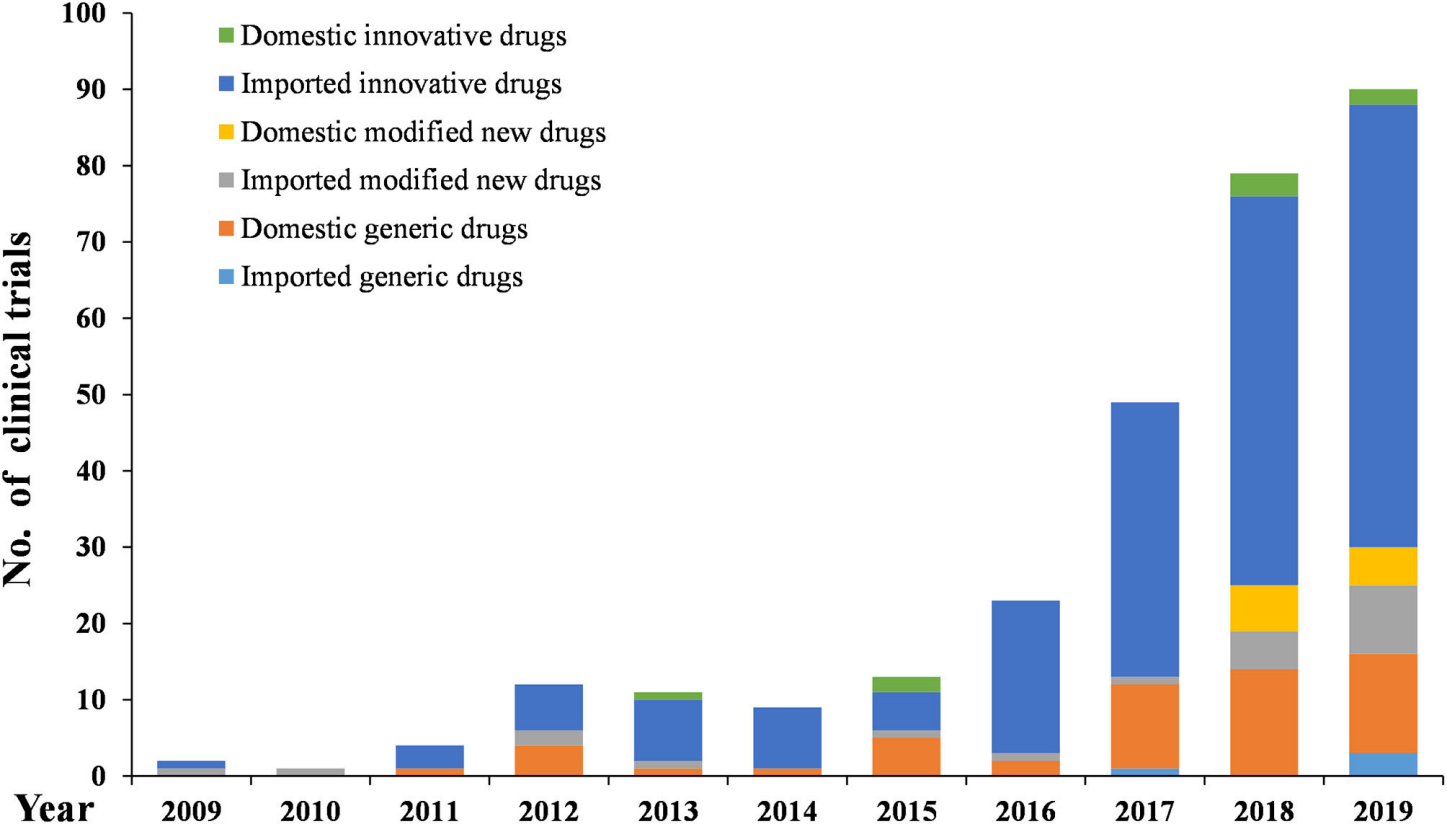
Development trends of different types of chemical drugs in pediatric clinical trials.

As Figures 6–8 show, in the 303 clinical trials on chemical drugs, 57 were on innovative drugs, accounting for 18.8%. However, only four trials on innovative drugs were sponsored domestically (domestic innovative drugs) (7.0%), far fewer than the 53 trials sponsored overseas (imported innovative drugs) (93.0%). From 2011 to 2019, trials on innovative drugs in mainland China showed an upward trend, with an average annual increase rate of 41.4% (*P* = 0.0038). The number of trials on innovative drugs has sharply increased increase in 2017 (500.0%) compared to 2016.

As for modified new drugs, 35 trials were conducted, accounting for 11.6%. Of these, 24 were sponsored domestically (domestic modified drugs) (68.6%), while another 11 trials were sponsored overseas (imported modified drugs) (31.4%). Before 2017, the number of trials on modified new drugs followed a steady trend while a drastic increased was reported in 2018, with an increase of 1,000.0% compared to 2017.

As for generic drugs, 211 clinical trials were conducted, accounting for 69.6%. Of these, 203 were sponsored domestically (domestic generic drugs) (96.2%), far more than the eight trials sponsored overseas (imported generic drugs) (3.8%). The number of trials on generic drugs also increased over time, with an average annual increase rate of 50.6% (*P* = 0.0003). Clinical trials on generic drugs began to increase significantly from 2016, with an increase of 185.7% compared to 2015. The increase rate slowed from 2017 to 2019 but retained an average rate of 47.0%.

#### Pediatric Drugs

After excluding trials on prophylactic biological products, the 388 remaining trials were further analyzed, and drugs indicated only for children were defined as pediatric drugs. Of the 388 clinical trials, 85 were on drugs especially designed for pediatric use, only accounting for 21.9%. The numbers of trials on various types of pediatric drugs are presented in Table 8. For the chemical drugs, pediatric clinical trials on psychotropic and antipsychotic drugs were the most common (19 trials, 44.2%), indicated for epilepsy and attention deficit hyperactivity disorder. This was followed by trials on anti-tumor agents (six trials, 14.0%) and anti-infective drugs (four trials, 9.3%). Trials on traditional Chinese medicines and natural medicines were mainly indicated for respiratory diseases (five trials, 41.7%). Most pediatric trials conducted on therapeutic biological products focused on growth hormones (27 trials, 90%). Overall, clinical trials on drugs for pediatric use showed an upward trend over time, with an average annual increase rate of 18.2% (*P* = 0.0003). The increase was especially significant in 2017, when the number of pediatric clinical trials increased by 10 compared to 2016, with an increase rate of 166.7%.

**Table 8:** Distribution of pediatric trials by drug type.

**Drug type**	**No. of clinical trials**	**No. of clinical trials on drugs for pediatric use**	**%**
Chemical drugs	303	43	14.2
Traditional Chinese medicines and natural medicines	16	12	75.0
Therapeutic biological products	69	30	43.5

#### Orphan Drugs

In accordance with the First *List of Rare Diseases* released in May 2018, and the *List of Reference on Rare Diseases in China*, clinical trials on drugs indicated for rare diseases were screened (8, 9). Of the 487 clinical trials, 45 were indicated for rare diseases with 29 orphan drugs involved. Of the 45 trials for rare diseases, 23 were indicated for hemophilia, accounting for 51.1%. Further analysis of the other 22 trials showed that the next most common indications were Dravet syndrome and pulmonary artery hypertension, with three trials each. This was followed by spinal muscular atrophy, Tourette syndrome, and Lennox-Gastaut syndrome, with two trials each. Of the 45 trials, 24 were conducted on chemical drugs while the other 21 were on therapeutic biological products. In terms of sponsorship, 20 trials were sponsored by domestic pharmaceutical companies, accounting for 44.4%, while the other 25 were sponsored by overseas companies, accounting for 55.6%. Clinical trials on orphan drugs also showed an upward trend over time, with an average annual increase rate of 27.1% (*P* = 0.0003).

### Sponsors and Institutions

#### Sponsor

**Figure 9:**
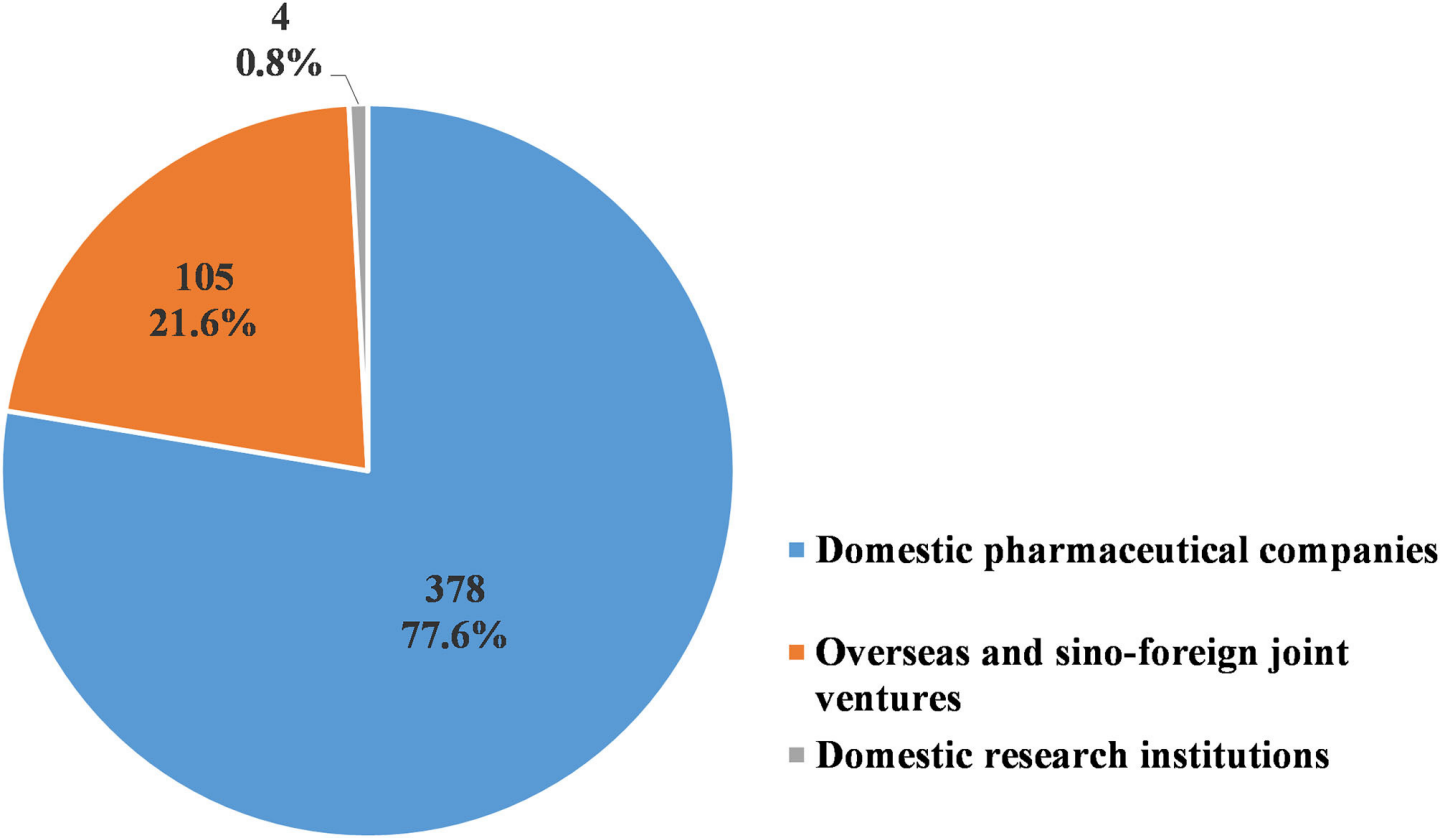
Sponsorship of pediatric clinical trials.

Of the 487 pediatric clinical trials conducted from 2009 to 2020, 378 trials (77.6%) were sponsored by pharmaceutical companies in China, while another 105 (20.3%) were sponsored by overseas and sino-foreign joint ventures. Only four trials (0.8%) were sponsored by research institutions in China (Figure 9). The domestic pharmaceutical companies most frequently conducted BE studies, with a total of 172 trials (45.5%). This was followed by Phase III trials, with 81 trials conducted (21.4%). The overseas and sino-foreign joint ventures most frequently conducted Phase III trials, with a total of 71 trials (67.6%). This was followed by Phase IV trials, with 14 trials conducted (15.2%) (Table 9).

**Table 9:** Phases of clinical trials by different sponsors.

**Sponsor**	**Phase I**	**Phase II**	**Phase III**	**Phase IV**	**BE study**	**Others**	**Total**
Domestic pharmaceutical companies	38	30	81	31	172	26	378
Overseas and sino-foreign joint ventures	4	6	71	16	3	5	105
Domestic research institutions	1	0	1	0	2	0	4

#### Leading Institutions

**Figure 10:**
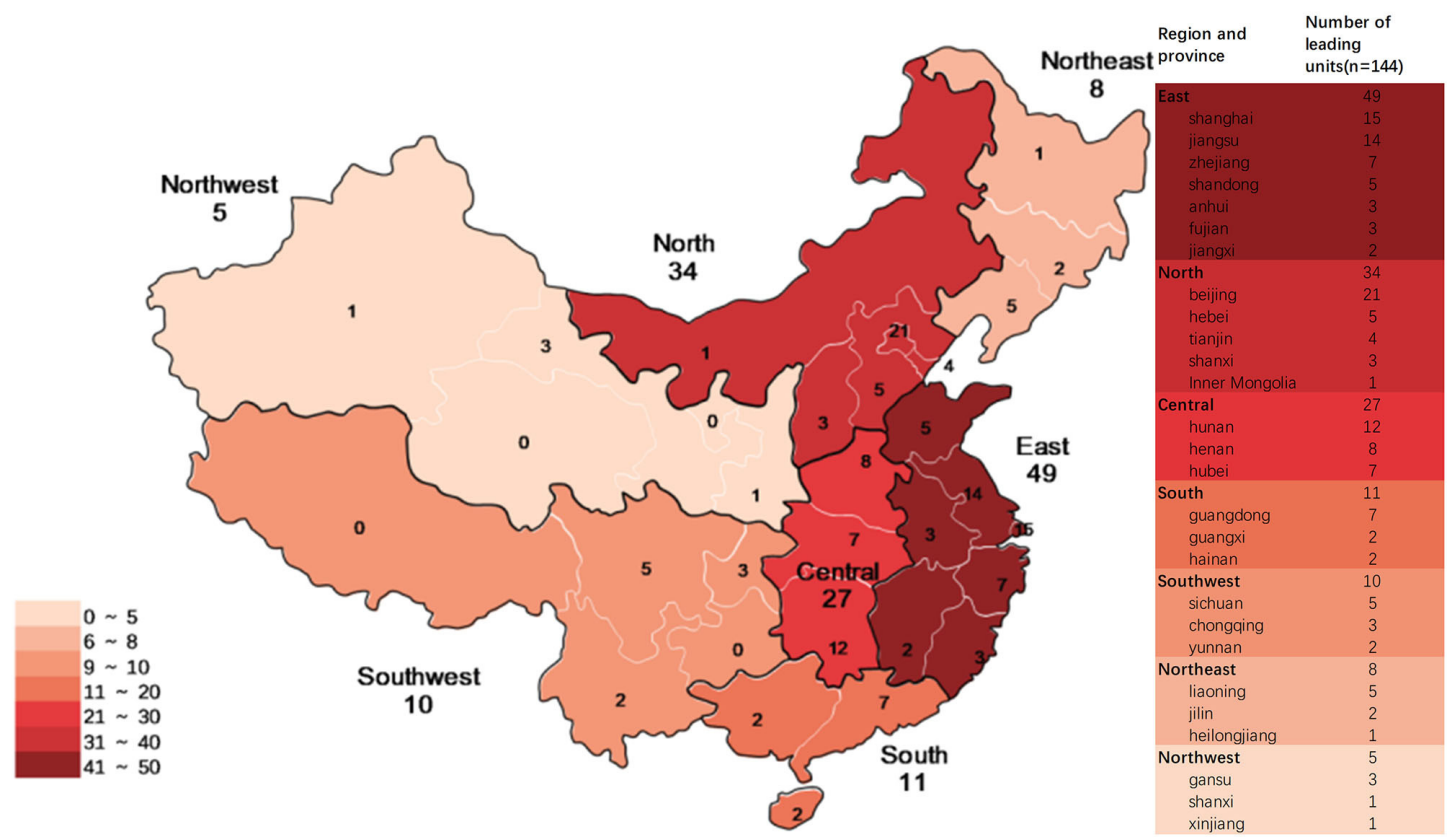
Distribution of leading unit institutions in mainland China.

From 2009 to 2019, 144 institutions participated in pediatric clinical trials in mainland China as leading unit institutions. Among these, 89 only participated in BE studies, and 17 (mainly local Centers for Disease Control and Prevention) only participated in studies on prophylactic biological products. East China had the highest number of leading unit institutions (49, 34.0%), North China had 34 leading unit institutions (23.6%), Central China had 27 (18.8%), South China had 11 (7.6%), Southwest China had 10 (6.9%), Northeast China had 8 (85.6%), and Northwest China had 5 (3.5%) (Figure 10).

## Discussion

Since children belong to a medically unique group, it is of great significance to protect their health. However, shortages of pediatric drugs are now a worldwide challenge, also experienced in China. Research on drug use in pediatric patients from 78 hospitals in seven locations in mainland China from 2013 to 2014 shows that drug types that can be used in pediatric patients only accounted for 31.04% of the drugs used, and drugs specifically designed for children only accounted for 0.7% (10). In addition, off-label drug use in the pediatric population in China is of great concern. A survey conducted by the Children's Hospital of Fudan University shows that 63.8% of pediatricians gave off-label prescriptions at least once and 92.45% of pharmacists dispensed off-label prescriptions at least once (11). Off-label drug use is common in the pediatric population at all ages. Owing to high costs and low profits, most Chinese pharmaceutical companies are reluctant to develop pediatric drugs and carry out the relevant trials. Therefore, low motivation for pediatric drug development and a lack of pediatric clinical trials may be the major causes of the above-mentioned challenges. Through a development process evaluation of the quantity and quality of pediatric drug clinical trials in China in the past decade, and considering the relevant policies issued by the Chinese government, we further analyzed and discussed the gains and losses of the development experience of pediatric drug clinical trials in China in the past decade.

### Pediatric Clinical Trials and Drug R&D Are Encouraged by National Policy

**Figure 11:**
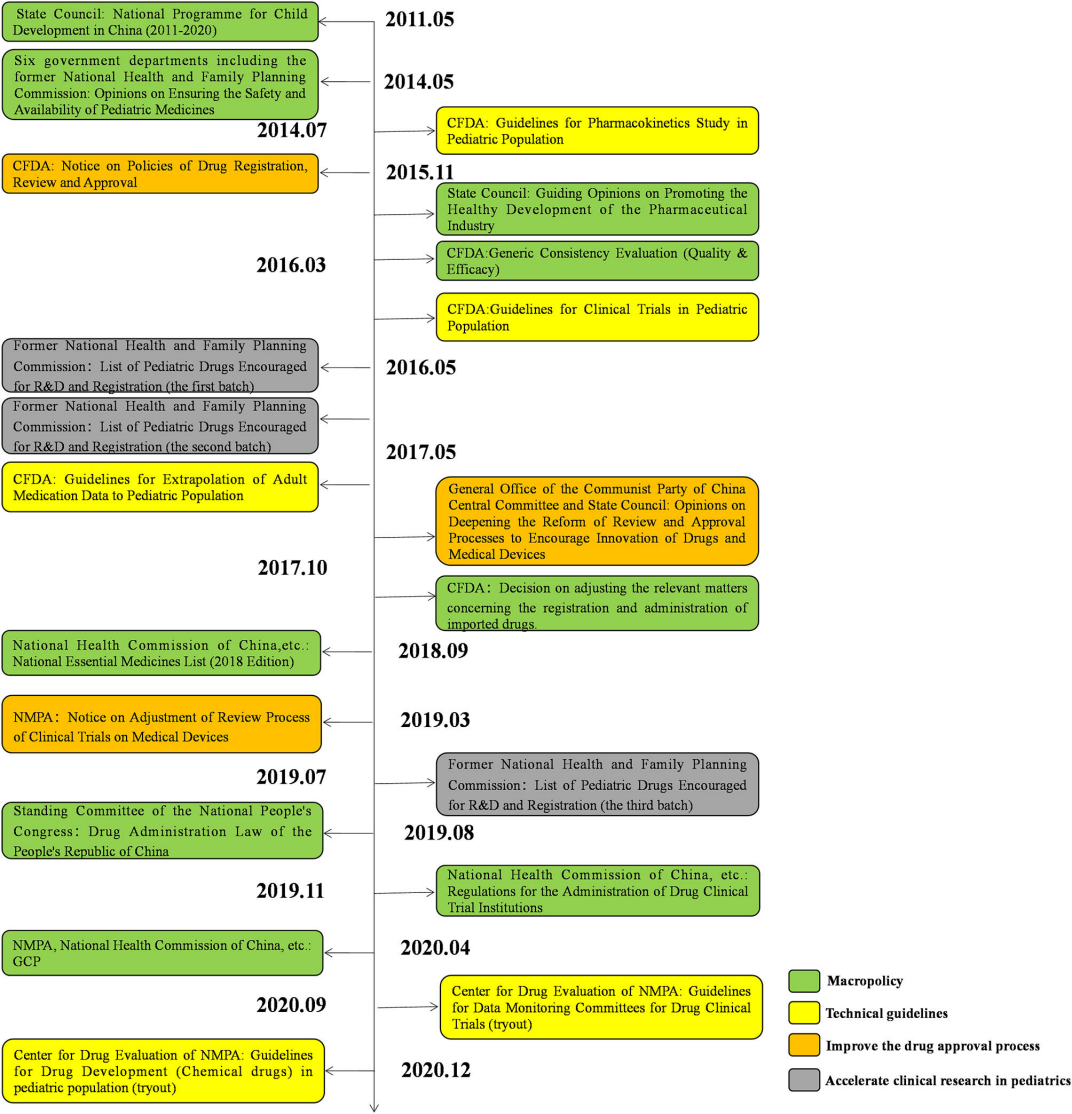
Policies and regulations.

To encourage the development of pediatric drugs and ensure children's access to such drugs, the Chinese government has launched a package of incentive policies and regulations over the past decade (Figure 11).

In 2011, the State Council issued the National Programme for Child Development in China (2011–2020), which proposed to “encourage the development and manufacture of drugs specifically for pediatric use.”(12) Since 2019, the Significant New Drugs Development Project under the 11, 12, and 13th Five-Year Plans has continuously supported the development of pediatric clinical trials. In 2014, six government departments, including the former National Health and Family Planning Commission, jointly issued Opinions on Ensuring the Safety and Availability of Pediatric Medicines, which recommended the establishment of special approval tracks for pediatric drugs and encouraged research and development (R&D) and clinical trials in the pediatric population, to ensure the availability of pediatric drugs (13). Moreover, in 2016 the State Council issued Guiding Opinions on Promoting the Healthy Development of the Pharmaceutical Industry. It suggested that it is of vital importance to develop appropriate dosage forms and new products based on the physiological characteristics of children to meet pediatric clinical needs (14). In 2017, the General Office of the Communist Party of China Central Committee and the State Council released Opinions on Deepening the Reform of the Review and Approval System to Encourage Innovation of Drugs and Medical Devices.

In this document a period of data exclusivity for clinical trials on pediatric drugs was proposed. The National Essential Medicines List, updated in 2018, added 22 pediatric drugs. Encouragingly, in the 2018 edition of the list, a separate category was introduced for pediatric drugs for the first time, which implies that more pediatric drugs will be incorporated into the list in the future (15). The Drug Administration Law of the People's Republic of China, amended in 2019, requires the government to take effective measures to encourage the R&D and innovation of pediatric drugs (16).

Against this backdrop, the National Medical Products Administration (NMPA), as the regulatory authority over drug development and clinical trials in China, has launched a series of policies to encourage the development of pediatric drugs and relevant trials (Figure 11). In 2013, the NMPA issued Opinions on Continuing the Reforms in Drug Review and Approval and Further Encouraging Pharmaceutical Innovation, which grants priority review to innovative and generic pediatric drugs with proper formulations and dosages based on the physiological characteristics of children (17). In 2014, the NMPA issued the first technical guideline on pediatric clinical trials, guiding investigators through how to conduct pharmacokinetic studies on the pediatric population (18). In 2015, the NMPA issued the Notice on Policies of Drug Registration, Review and Approval, which proposed the establishment of a special review track for pediatric drugs with an accelerated review process (19). In 2016, the former National Health and Family Planning Commission, CFDA, and Ministry of Industry and Information Technology jointly issued the List of Pediatric Drugs Encouraged for R&D and Registration (the first batch). Since 2016, three batches of the list have been released, covering a total of 105 pediatric drugs (20–22). In 2018, the NMPA released the Notice on Adjustment of Review Process of Clinical Trials on Medical Devices, replacing the former qualification certification with a record-keeping management system. (23) In the same year, the Measures for Data Protection for Clinical Trials (Provisional) was issued, which granted a 6-year data exclusivity period to pediatric drugs to protect the interests of innovators (24). In 2019, the NMPA issued the Regulations for the Administration of Drug Clinical Trial Institutions, which officially changed the accreditation of drug clinical trial institutions to record-keeping management (25). Apart from government policies, a series of technical guidelines have been established since 2014, including the Guidelines for Pharmacokinetics Study in Pediatric Population, Guidelines for Clinical Trials in Pediatric Population, Guidelines for Extrapolation of Adult Medication Data to Pediatric Population, and Guidelines for Drug Development (Chemical drugs) in Pediatric Population (Tryout). These guidelines systematically analyzed the crucial problems existing in pediatric clinical trials and provide technical support for pediatric drug development (18, 26–28).

### Increasing Pediatric Drug Clinical Trials and International Cooperation

Stimulated by these incentive policies, the number of pediatric clinical trials continued to increase from 2009 to 2019, with an average annual increase rate of 44.7%. The increase was especially significant after 2016, when a series of polices encouraging the development and approval of pediatric drugs were launched, various technical guidelines were released, and the record-keeping management system for clinical trial institutions and the default licensing system of clinical trials were implemented was adopted. Pediatric clinical trials conducted from 2016 to 2019 accounted for 73.7% of all the pediatric trials conducted over the past decade.

Since the ethical requirements and standards of pediatric clinical trials varied across different regions of China, it has been difficult to conduct multi-center clinical trials. In 2017, Opinions on Deepening the Reform of Review and Approval Processes to Encourage Innovation of Drugs and Medical Devices was issued, which requires that, once a multi-center clinical trial planned to initiate in China has obtained ethical approval from one leading unit institution, other member institutions shall conduct the review according to the review opinion to avoid repeated reviews (16). This has led to more multi-center clinical trials, and the data we collected confirmed this. According to our statistics, 43.0% of the pediatric clinical trials conducted after the release of the document have been multi-center ones. Subsequently, the NMPA issued the Decision on Adjusting the Relevant Matters Concerning the Registration and Administration of Imported Drugs, which lifted some of the restrictions on international multi-center clinical trials planned to initiate in China. The document stipulates that, when international multi-center clinical trials are completed, the sponsors can submit new drug applications in China directly (29). This greatly encourages foreign pharmaceutical companies to conduct clinical trials synchronously in China, which provides more opportunities for China to participate in international multi-center pediatric clinical trials. Our study shows that international multi-center pediatric trials began to increase significantly in 2017. The average number of international multi-center trials conducted from 2017 to 2019 is 14.1 times higher than that in the preceding years.

### The Quality of Pediatric Drug Clinical Trials Needs to Be Further Improved

China has made remarkable progress in conducting pediatric clinical trials over the past decade. However, there remains a scope for improvement with respect to trial quality, as there are still deficiencies in pediatric trials in mainland China. These are mainly reflected in the following aspects.

#### Lack of Innovation, Rather Concentrated Research Areas, and Prevalent Repetitive R&D

Owing to the high costs of drug development and the relatively small scale of domestic pharmaceutical companies compared to some international ones, domestic pharmaceutical companies often lack the funding and capability to invest in innovative drugs. With the support of incentive policies, the R&D of innovative drugs in China has progressed, which is particularly obvious in areas like small-molecule targeted drugs for tumor treatment and immune checkpoint inhibitors. However, little progress has been made in pediatric drugs since it is harder to convert active pharmaceutical ingredients to final pharmaceutical products and takes longer to see investment return for pediatric medicines. Therefore, most domestic pharmaceutical companies still focus on pediatric generics with mature markets, as they require a lower budget and provide a quicker investment return.

Of the 487 pediatric clinical trials, BE studies accounted for 36.3% and only four of them were sponsored by overseas pharmaceutical companies. This directly reflects the release of the Generic Consistency Evaluation (Quality & Efficacy) by the State Council in 2016. This evaluation requires BE studies to be conducted on all solid-dosage oral pharmaceuticals to test the consistency between generics and the original product. Solid-dosage pharmaceuticals without BE studies can no longer be registered (30). Thus, since 2016, the number of BE studies has increased dramatically. In 2019, BE studies accounted for over half of all the pediatric trials, indicating that generics play a dominant role in pediatric trials.

Our study shows that, of the 303 pediatric trials on chemical drugs, 211 were for generics and 203 of these (96.2%) were sponsored by domestic pharmaceutical companies. In contrast, trials on innovative drugs sponsored by domestic companies were rare. In the 57 trials on innovative drugs, only four (7.0%) were sponsored by domestic companies, far fewer than the 53 trials on imported innovative drugs. In recent years, clinical trials on popular innovative drugs, such as JAK kinase inhibitors, monoclonal antibodies, gene splicing and modification drugs, and DNA repair drugs were carried out in China overwhelming by overseas companies. Consider monoclonal antibodies as an example, there were seven related trials, of which only one was sponsored by a company in mainland China while the rest were all sponsored and developed by overseas companies. Of the 25 trials on orphan drugs, nine were sponsored by domestic companies, of which only two involved modified new drugs while the rest all involved generics. In contrast, of the 14 trials sponsored overseas, only one was on a generic drug while the others were all on innovative or modified drugs.

Overall, pediatric clinical trials in China mainly focus on infectious, nervous, respiratory, and endocrine diseases and vaccines, research areas that are relatively mature with less R&D risk. In contrast, there were few trials on cancer, immune diseases, or cardiovascular diseases, research areas with no R&D model to follow and thus with a higher risk. However, cancer, immune diseases, and cardiovascular diseases are usually the main causes of death for children in China. (31) Excessive clinical trials concentrated in just a few therapeutic areas result in repeated research studies and limited types of new drugs. Our study found that only four different drugs were involved in the 27 trials on growth hormone, and four others in the 24 trials on antihistamines, giving an average of six to seven clinical trials investigating each drug. In addition, the average number of trials investigating each drug for antivirals, antiepileptics, and coagulation factors was 2.5–3. A total of 21 clinical trials on different dosage forms of montelukast sodium were registered, and as many as 30 trials investigated tenofovir-related single or compound preparations. Too many repeated and unnecessary clinical studies decrease the innovative capacity of pediatric trials in China, eroding the development environment for innovative drugs.

Notably, although there has been no breakthrough for domestic pharmaceutical companies in innovative drugs, the number of modified new drugs for pediatric use has increased significantly since 2018. The number of trials on modified new drugs for pediatric use in 2018 and 2019 was 14, while the total number of such trials before 2018 was only nine. Modified new drugs improve or optimize the formulation, structure, or dosage form of original products, or add new indications. Compared to innovative drug trials, modified new drug trials, the development of which is also supported by national policies, focus more on “superiority”. Modified new drugs have lower risks, higher success rates, higher investment returns, and longer product life cycles. Therefore, at the current stage, developing modified new drugs may be the best choice for companies mainly relying on generics and lacking innovative capacity.

#### Less Standardized Clinical Trials Design

At present, pediatric clinical trials in China follow the principles and standards of adult trials, regardless of trial design or methodology. However, children have different physiological characteristics at various development stages, which indicates that pediatric trials, with higher potential safety risks, cannot be the conducted in the same manner as those of adults. According to the Annual Report for National Adverse Drug Reaction Monitoring (2019), there were 1.51 million cases of adverse drug reactions, of which children under the age of 14 accounted for 10.8% (32). It is therefore urgent to grant more attention to pediatric clinical trials from both ethical and scientific perspectives. Trial-related injury insurance can effectively lower safety risks and improve trial quality; however, the situation regarding this aspect in China is not optimistic.

Trial-related insurance plays an important role in protecting the interests of participants. It also serves as an important risk-sharing mechanism for sponsors and helps to avoid unnecessary disputes between participants, hospitals, and ethical committees. Moreover, trial-related insurance can reassure parents, alleviating concerns regarding the potential risks of clinical trials, which helps with the recruitment of child subjects. To date, China has not made any compulsory provisions that require sponsors to provide insurance. The newly revised Good Clinical Practice, published in 2020, only requires that “Sponsors should provide trial-related legal and economic insurance or guarantees to investigators and institutions” (33). The data we collected show that, for the 487 pediatric clinical trials, the overall insurance coverage rate was 44.8%, much lower than that of anti-tumor clinical trials (79.8%) (34). The insurance coverage rate of clinical trials sponsored by overseas companies (87.7%) was much higher than that of domestically sponsored trials (32.8%), and the coverage rate of international multi-center trials (88.5%) was also much higher than that of domestic trials (38.5%). The relatively low insurance coverage rate in China results from the absence of relevant regulations, weak public awareness of rights protection, and an immature commercial insurance industry, among other factors. However, the insurance rate of domestic pediatric trials has been increasing over the past decade, with an average rate of 42.7% from 2017 to 2019. In 2020, of the 11 domestic pediatric trials, eight have provided insurance, with a coverage rate of 72.7%. With the introduction of more relevant laws and improvements in public awareness of rights protection, the gap in trial-related insurance coverage between China and the international community can be narrowed further.

#### Uneven Distribution of Pediatric Clinical Trials

In addition, with government policies supporting pediatric drug development and clinical trials, the number of clinical institutions with pediatrics is also increasing. By December 2019, 158 clinical institutions had been approved to conduct pediatric trials. There was a surge in the number of clinical institutions with pediatric approval from 2017 to 2019. During this period, 91 new institutions were approved, accounting for 57.6% of all approvals. The surge may be owing to the issue of the Generic Consistency Evaluation by the CFDA in 2016.

Our study found that 61.8% of the pediatric clinical institutions only carried out BE studies. Pediatric clinical institutions in China still face two main challenges: First, there remains an insufficient number of clinical institutions with pediatric capacity, accounting for only 17.7% of all the clinical institutions by December 2019. Second, similar to the distribution of leading unit institutions, the distribution of clinical institutions with pediatrics is imbalanced, with most institutions in East China and North China, followed by Central China, South China and Southwest China, Northwest China, and Northeast China (in descending order). This difference does not reflect demographic factors or patient distributions, instead, indicate the uneven distribution of clinical medical resources in China. Thus, it is important for policy makers to coordinate the distribution of medical resources while acknowledging the leading role of some areas.

#### Shortage of Pediatric Drug R&D, Priority Review, and Approval Policies Have Not Yet Fully Played Its Role

Clinical trials are the basis for drug marketing, and a highly efficient review and approval system is an important guarantee for the successful marketing of pediatric drugs. With the rapid development of pediatric clinical trials in China and the implementation of relevant priority review and approval policies, the speed by which pediatric drugs are marketed in China has increased in recent years. However, compared with Japan, Europe, United States, and other regions, there is still further improvement to be made. Taking pediatric drugs marketed through the priority review and approval policy as an example, in the past five years, a total of 29 pediatric drugs, accounting for 8.5% among all priority review and approval drugs (343), have been rapidly approved in China for marketing through this policy. (35) In the past two decades, Japan has passed a similar policy named “public knowledge-based applications” *(Kouchi-shinsei* in Japanese*)*, and 74 pediatric drugs have been approved under this policy, accounting for 33.8% of all approved drugs (219) in the project, which is still higher than that (8.5%) in China. (36) EU data show that 25% of all new medicines released in the EU were aimed for children during 2007–2017 (37). Although there are no specific data on the marketing of pediatric drugs in the US, according to the statistics of the Food and Drug Administration, under the strong promotion of the *Best Pharmaceuticals for Children Act* and the *Pediatric Research Equity Act*, pediatric clinical trials based on the original trials have been re-conducted on as many as 792 drugs, and the pediatric sections on package inserts of 854 drugs have been revised. (38) Correspondingly, the 487 clinical trials included in our research involved only 198 drugs. However, we should also note that as increasing support has been provided by the Chinese government to the pediatric drug accessibility, research and development on and review and approval of pediatric drugs have been accelerated in recent years. In 2020, the NMPA approved a total of 26 pediatric drugs, with a yearly increase of 36.8%. In the first half of 2021, 14 pediatric drugs have been approved for marketing, and dozens of pediatric drugs and varieties for adding indications or usage and dosage are under review, among which 22 varieties have been included in the priority review and approval sequence (39).

## Limitations

The raw data used in this study were derived from the only mandatory clinical trial registry platform in mainland China and we conducted a systematic review of the pediatric clinical trials registered on this platform over the past decade. However, the study also has some limitations. Although it is compulsory for sponsors to register information regarding their clinical trials on the platform and the information submitted are reviewed and verified by the CFDA, which ensures data integrity and reliability to a large extent, there was no such requirement before September 2013. That is, clinical trials completed before 2013 may not be included in the platform database. In addition, despite being required to submit the initial registration at least 30 days before the first subject is enrolled, the submission time of trials can differ. Therefore, this study defined the initial year of a trial by the date of its first ethical review, which differs from other similar papers in China.

## Conclusions and Outlook

It was proposed in the National Programme for Child Development in China (2011–2020) that children are the future of mankind, and child health is the foundation of national health and an important guarantee for sustainable economic and social development (9). Therefore, the Chinese government has been committed to promoting the development of pediatric drugs. Over the past decade, China has made constant progress in improving the R&D environment for pediatric drugs and conducting more pediatric trials. However, there remains a wide gap in pediatric drug development between China and developed countries, and limitations such as study duplication, a lack of innovation, and poor research design remains a challenge in mainland China. Currently, China's national policies are continuously tilting in favor of innovative drugs. Influenced by medical reform policies such as volume-based procurement, generic consistency evaluation, and healthcare cost control, the profitability of generic drugs is decreasing annually and the transformation and upgrading of the pharmaceutical industry is imminent. Since the NMPA joined the ICH, China has taken actions to further harmonize with international regulatory standards to conduct safer, more effective, and higher quality clinical drug trials. Thus, it can be believed that, with improvements in regulatory policies and the innovative capacity of the pharmaceutical industry, China, as the world's second largest pharmaceutical market, will contribute more substantially to the global development of pediatric drugs.

## Data Availability Statement

The original contributions presented in the study are included in the article/supplementary material, further inquiries can be directed to the corresponding authors.

## Author Contributions

W-WW, QW, and JX designed, planned, and led the study. W-WW, XJ, and HW undertook the literature search and with FC applied the eligibility criteria. W-WW, QD, G-dZ, and ML retrieved the clinical information from the Chinese Clinical Trials Registry and Information Transparency Platform. W-WW, S-sW, M-mN, and Q-qL undertook data extraction. W-WW, XJ, and HW designed and applied the statistical methods utilized. W-WW, QW, and HW wrote the original draft of the paper. All authors participated in the preparation, review and editing process of this paper, and have approved the final article.

## Funding

This work was supported by the Hospital Pharmacy Foundation of Nanjing Pharmaceutical Association–Changzhou SiYao Pharmaceuticals (2020YX024) and the Specially-Appointed Medical Expert Project of the Jiangsu Commission of Health (2019).

## Conflict of Interest

The authors declare that the research was conducted in the absence of any commercial or financial relationships that could be construed as a potential conflict of interest.

## Publisher's Note

All claims expressed in this article are solely those of the authors and do not necessarily represent those of their affiliated organizations, or those of the publisher, the editors and the reviewers. Any product that may be evaluated in this article, or claim that may be made by its manufacturer, is not guaranteed or endorsed by the publisher.

## Abbreviations

CCTR and ITP: Chinese Clinical Trials Registry and Information Transparency Platform
Chi CTR: Chinese Clinical Trial Registry
NMPA: National Medical Products Administration
UN: United Nations
CFDA: China Food and Drug Administration
BE: Bioequivalence
R&D: Research and Development
ICH: International Council for Harmonization of Technical Requirements for Pharmaceuticals for Human Use.

## References

[1] White Paper on the Investigation of Pediatric Drug Use Safety. (2016). Available online at: https://www.ruiwen.com/gongwen/diaochabaogao/99076.html (accessed April 30, 2020).

[2] Li J Yan K Kong YT Ye XF Ge MM Zhang CF. A cross-sectional study of children clinical trials registration in the world based on ClinicalTrials.gov establishment. Chin J Evid Based Pediatr. (2016) 11:3–7. 10.3969/j.issn.1673-5501.2016.01.002

[3] Zhang GD Yang Y Zhao RL. Characteristics Analysis of Pediatric Drug Clinical Trials Registered in Chinese Clinical Trial Registry. China Pharmacy. (2020) 31:2055–60. 10.6039/j.issn.1001-0408.2020.17.02

[4] Hao GX Yuan XX Guo W Quan XY Qi XJ Wang TY Zhao W. Paediatric drugs trials in China. BMJ Paediatrics Open. (2020) 4:e000618. 10.1136/bmjpo-2019-000618 32342015 PMC7173953

[5] National Health Commission of the People's Republic of China. Chinese National Formulary. (2010). Available online at: http://www.nhc.gov.cn/cms-search/xxgk/getManuscriptXxgk.htm?id=45875 (accessed April 30, 2020).

[6] National Medical Products Administration. Provisions for Drug Registration. (2020). Available online at: https://www.nmpa.gov.cn/xxgk/fgwj/bmgzh/20200330180501220.html (accessed April 30, 2020).

[7] National Medical Products Administration. Reform Scheme of the Classification System for Registration of Chemical Drugs. (2016). Available online at: https://www.nmpa.gov.cn/yaopin/ypggtg/ypqtgg/20160309151801706.html (accessed April 30, 2020).

[8] National Health Commission of the People's Republic of China. First List of Rare Diseases. (2018). Available online at: http://www.nhc.gov.cn/cms-search/xxgk/getManuscriptXxgk.htm?id=393a9a37f39c4b458d6e830f40a4bb99. (accessed April 30, 2020).

[9] List of Reference on Rare Diseases in China. (2018). Available online at: http://www.cord.org.cn/. (accessed April 30, 2020).

[10] Yiyun LI Zhi'ang WU Xin HU. Analysis of drug use in pediatric patients from 78 sample hospitals in 7 regions of China from 2013 to 2014. China Pharmacy. (2016) 27:4058–61. 10.6039/j.issn.1001-0408.2016.29.07

[11] Mei M Wang L Liu E Li Z Guo Z Zhang X Xu H. Current practice, management and awareness of pediatric off-label drug use in China-A questionnaire based cross sectional survey. Chin J Evid Based Pediatr. (2017) 12:289–94. 10.1186/s12887-019-1664-7 31409339 PMC6691537

[12] People's Republic of China of State Council National Health Commission of the People's Republic of China (NHCPRC). National Programme for Child Development in China (2011–2020). (2011). Available online at: http://www.nhc.gov.cn/fys/s7900/201108/0990d658c2a74a7dae622684d24b2985.shtml. (accessed April 30, 2020).

[13] National Health Commission of the People's Republic of China. Opinions on Ensuring the Safety and Availability of Pediatric Medicines. (2014). Available online at: http://www.nhc.gov.cn/yaozs/s3581/201405/e51354d631944fa68aac0c4d9585f291.shtml (accessed April 30, 2020).

[14] People's Republic of China of State Council. Guiding Opinions on Promoting the Healthy Development of the Pharmaceutical Industry. (2016). Available online at: http://www.gov.cn/zhengce/content/2016-03/11/content_5052267.htm (accessed April 30, 2020).

[15] People's Republic of China of State Council. Opinions on Deepening the Reform of the Review and Approval System to Encourage Innovation of Drugs and Medical Devices. (2017). Available online at: http://www.gov.cn/zhengce/2017-10/08/content_5230105.htm (accessed April 30, 2020).

[16] People's Republic of China of State Council. Drug Administration Law of the People's Republic of China. (2019). Available online at: http://www.gov.cn/xinwen/2019-08/26/content_5424780.htm (accessed April 30, 2020).

[17] National Medical Products Administration. Opinions on Continuing the Reforms in Drug Review and Approval and Further Encouraging Pharmaceutical Innovation. (2013). Available online at: https://www.nmpa.gov.cn/zwgk/xwfb/20130226195001883.html (accessed April 30, 2020).

[18] National Medical Products Administration. Guidelines for Pharmacokinetics Study in Pediatric Population. (2014). Available online at: https://www.nmpa.gov.cn/directory/web/nmpa/xxgk/fgwj/gzwj/gzwjyp/20140711112001393.html (accessed April 30, 2020).

[19] National Medical Products Administration. Notice on Policies of Drug Registration, Review and Approval. (2015). Available online at: https://www.nmpa.gov.cn/directory/web/nmpa/xxgk/ggtg/qtggtg/20151111120001229.html (accessed April 30, 2020).

[20] National Health Commission of the People's Republic of China. List of Pediatric Drugs Encouraged for R&D and Registration (the first batch). (2016). Available online at: http://www.nhc.gov.cn/yaozs/s3581/201605/b0ea217312314c5098d905094f7e67ee.shtml (accessed April 30, 2020).

[21] National Health Commission of the People's Republic of China. List of Pediatric Drugs Encouraged for R&D and Registration (the second batch). (2017). Available online at: http://www.nhc.gov.cn/yaozs/s3581/201705/b9874725a6a04e2ebbc8a969a4604609.shtml (accessed April 30, 2020).

[22] National Health Commission of the People's Republic of China. List of Pediatric Drugs Encouraged for R&D and Registration (the third batch). (2019). Available online at: http://www.nhc.gov.cn/yaozs/s7656/201908/9a10b2382fe94d84817d9044d90dda15.shtml (accessed April 30, 2020).

[23] National Medical Products Administration. Notice on Adjustment of Review Process of Clinical Trials on Medical Devices. (2019). Available online at: https://www.nmpa.gov.cn/xxgk/ggtg/qtggtg/20190401164701503.html (accessed April 30, 2020).

[24] National Medical Products Administration. Measures for Data Protection for Clinical Trials (Provisional). (2018). Available online at: https://www.nmpa.gov.cn/directory/web/nmpa/xxgk/zhqyj/zhqyjyp/20180426171801468.html (accessed April 30, 2020).

[25] National Medical Products Administration. Regulations for the Administration of Drug Clinical Trial Institutions. (2019). Available online at: https://www.nmpa.gov.cn/xxgk/ggtg/qtggtg/20191129174401214.html (accessed April 30, 2020).

[26] National Medical Products Administration. Guidelines for Clinical Trials in Pediatric Population. (2016). Available online at: https://www.nmpa.gov.cn/xxgk/ggtg/qtggtg/20160307164401912.html (accessed April 30, 2020).

[27] National Medical Products Administration. Guidelines for Extrapolation of Adult Medication Data to Pediatric Population. (2017). Available online at: https://www.nmpa.gov.cn/directory/web/nmpa/xxgk/ggtg/qtggtg/20170518163201802.html (accessed April 30, 2020).

[28] Center for Drug Evaluation National Medical Products Administration. Guidelines for Drug Development (Chemical drugs) in pediatric population (tryout). (2020). Available online at: http://www.cde.org.cn/news.do?method=largeInfo&id=b40a4d0fd292259b (accessed January 30, 2021).

[29] National Medical Products Administration. Decision on adjusting the relevant matters concerning the registration and administration of imported drugs. (2017). Available online at: https://www.nmpa.gov.cn/yaopin/ypjgdt/20171010213601206.html (accessed April 30, 2020).

[30] People's Republic of China of State Council. Generic Consistency Evaluation (Quality & Efficacy). (2016). Available online at: http://www.gov.cn/zhengce/content/2016-03/05/content_5049364.htm (accessed April 30, 2020).

[31] Wu W Tang Z Chen J Gao Y. Pediatric drug development in China: Reforms and challenges. Pharmacological Research. (2019) 148:104412. 10.1016/j.phrs.2019.104412 31491470

[32] Center for Drug Reevaluation National Medical Products Administration. National Center for ADR Monitoring, China. Annual Report for National Adverse Drug Reaction Monitoring (2019). (2020). Available online at: http://www.cdr-adr.org.cn/tzgg_home/202004/t20200410_47300.html (accessed April 30, 2020).

[33] National Medical Products Administration National Health Commission of the People's Republic of China. Good Clinical Practice. (2020). Available online at: https://www.nmpa.gov.cn/xxgk/ggtg/qtggtg/20200426162401243.html (accessed April 30, 2020).

[34] Li N Huang HY Wu DW Yang ZM Wang J Wang JS . Changes in clinical trials of cancer drugs in mainland China over the decade 2009-18: a systematic review. Lancet Oncol. (2019) 20:e619–26. 10.1016/S1470-2045(19)30491-7 31674320

[35] Center for drug evaluation NMPA Report on drug Evaluation in 2020. (2021). Available online at: http://www.cde.org.cn/news.do?method=largeInfo&id=cb377a202489c901 (accessed Aug 16, 2021).

[36] Maeda H Fukuda Y Uchida M. Assessment of drugs approved by public knowledge-based applications (*Kouchi-shinsei*) during the last two decades in Japan. Clin Pharmacol Ther. (2021). 10.1002/CPT.2332 34110632 PMC8518418

[37] European Parliament and the Council of the European Union State of Paediatric Medicines in the EU: 10 Years of the EU Paediatric Regulation. (2017). Available online at: https://ec.europa.eu/health/sites/default/files/files/paediatrics/docs/2017_childrensmedicines_report_en.pdf (accessed Aug 16, 2021).

[38] U.S. FOOD&DRUG ADMINISTRATION.New Pediatric Labeling Information Database. (2021). Available online at: https://www.accessdata.fda.gov/scripts/sda/sdNavigation.cfm?sd=labelingdatabase (accessed Aug 16, 2021).

[39] National Medical Products Administration.Jiao Hong emphasize in the drug trial center on-site office to commit to hard soul to do the children's medication review and approval, NMPA. (2021). Available online at: https://www.nmpa.gov.cn/yaowen/ypjgyw/20210726213505143.html (accessed Aug 16, 2021).

